# Equity in Maternal Health in South Africa: Analysis of Health Service Access and Health Status in a National Household Survey

**DOI:** 10.1371/journal.pone.0073864

**Published:** 2013-09-06

**Authors:** Njeri Wabiri, Matthew Chersich, Khangelani Zuma, Duane Blaauw, Jane Goudge, Ntabozuko Dwane

**Affiliations:** 1 Epidemiology and Strategic Information Unit, Human Sciences Research Council, Pretoria, South Africa; 2 Centre for Health Policy, School of Public Health, University of the Witwatersrand, Johannesburg, South Africa; 3 International Centre for Reproductive Health, Department of Obstetrics and Gynaecology, Ghent University, Gent, Belgium; UCL Institute of Child Health, University College London, United Kingdom

## Abstract

**Background:**

South Africa is increasingly focused on reducing maternal mortality. Documenting variation in access to maternal health services across one of the most inequitable nations could assist in re-direction of resources.

**Methods:**

Analysis draws on a population-based household survey that used multistage-stratified sampling. Women, who in the past two years were pregnant (1113) or had a child (1304), completed questionnaires and HIV testing. Distribution of access to maternal health services and health status across socio-economic, education and other population groups was assessed using weighted data.

**Findings:**

Poorest women had near universal antenatal care coverage (ANC), but only 39.6% attended before 20 weeks gestation; this figure was 2.7-fold higher in the wealthiest quartile (95%CI adjusted odds ratio = 1.2–6.1). Women in rural-formal areas had lowest ANC coverage (89.7%), percentage completing four ANC visits (79.7%) and only 84.0% were offered HIV testing. Testing levels were highest among the poorest quartile (90.1% in past two years), but 10% of women above 40 or with low education had never tested. Skilled birth attendant coverage (overall 95.3%) was lowest in the poorest quartile (91.4%) and rural formal areas (85.6%). Around two thirds of the wealthiest quartile, of white and of formally-employed women had a doctor at childbirth, 11-fold higher than the poorest quartile. Overall, only 44.4% of pregnancies were planned, 31.7% of HIV-infected women and 68.1% of the wealthiest quartile. Self-reported health status also declined considerably with each drop in quartile, education level or age group.

**Conclusions:**

Aside from early ANC attendance and deficiencies in care in rural-formal areas, inequalities in utilisation of services were mostly small, with some measures even highest among the poorest. Considerably larger differences were noted in maternal health status across population groups. This may reflect differences between these groups in quality of care received, HIV infection and in social determinants of health.

## Introduction

South Africa is one of the most inequitable countries worldwide, by almost any measure. The wealthiest 10% of the population, for example, account for more than half of the country’s income [1]. Child mortality is twice as high in the rural Eastern Cape compared with the more urban Western Cape, and four times higher for black than for white individuals [2]. In regards to maternal health, institutional-level maternal mortality rates (MMR) vary considerably between provinces, from 84.9 maternal deaths per 100,000 live births in the Western Cape to levels of 289.1 maternal deaths per 100,000 live births in the Free State Province [3]. In five of the 52 districts in the country, the MMR is 300 or more, while it is below 100 in 10 other districts [3].

The Saving Mothers report for 2008–2010, a triennial confidential enquiry into maternal deaths, shows that maternal mortality levels have increased compared to previous trienniums, across all levels of health care [3]. These deaths are mostly due to HIV and other non-pregnancy related infections (41%), obstetric haemorrhage (14%) and hypertension (14%). Suboptimal care was noted in two thirds of these deaths, which stemmed largely from deficiencies in the knowledge and skills of health providers, and from poor functioning of the health system.

Deficiencies in access to maternal health services also make a critical contribution to maternal deaths. In the most recent Demographic and Health Survey (DHS) in 2003, 8% of women had not attended antenatal care and 9% delivered without a skilled attendant. Notably, only 56% of women in the survey had at least four antenatal care (ANC) visits and 46% had their first visit before 20 weeks. Aggregated figures at national level hide substantial differentials in access across the country. For example, in 2003, 85% of white women compared to 44% of black women attended ANC before 20 weeks of pregnancy, and only 13% of rural women delivered with a doctor, half the national figure [4].

Despite some key advances in maternal health research in South Africa, such as having district-level figures regarding HIV prevalence in pregnant women [5], little detailed information is available about the distribution of access to maternal health services across the country’s population groups. By examining variation in service utilisation (including amount of services received and timing of use) within a previous national household survey, this article aims to describe inequities in access to maternal health services in South Africa. The study primarily investigates differences in access according to socio-economic status, but also assesses the geographical distribution of services and the how access varies by factors such as rural-urban dwelling, race and HIV status. The study also aims to examine the influence of socio-economic status and these other factors on self-assessed maternal health status. Thematic maps of geographical distribution of skilled birth attendance (SBA) and ANC visits provide details of inequalities at district level. Finally, levels of maternal inequality in South Africa are examined using absolute and relative indicators of inequality.

## Methods

### Survey Sampling, Field and Laboratory Procedures

This paper is a sub-analysis of the third South African National HIV Prevalence, Incidence, Behaviour and Communication Survey [6]; data available from http://curation.hsrc.ac.za/Datasets-PFAJLA.phtml. This cross-sectional population-based household survey was conducted from May 2008 to March 2009, using multistage stratified sampling by: province; locality (urban formal, urban informal, rural formal including commercial farms, and rural informal or tribal areas); and predominant racial groups. Sampling frames were based on enumerator areas (EA) used in the national census, updated to reflect changes in the socio-demographic profile of the country since 2001. A total of 1000 EAs were selected from a database of 86,000 EAs as the primary sampling units; 15 households within each EA constituted the secondary sampling unit (15,000 households) and four eligible individuals selected within households formed the final sampling unit. Only one person in each age group (0–1, 2–11, 12–14, 15–24, 25 or more years) was selected in each household. If a household contained two or more persons in an age category, such a two children under the age of two years, a Kish table was used for selecting one person in each age group per household [7]. Any person who slept in the household on the night preceding the survey (including visitors) was considered a household member. All household members in the selected households were eligible to participate, including those living in hostels, but people staying in educational institutions, old-age homes, hospitals and uniformed-service barracks, as well as homeless people, were excluded from the survey.

Study activities were approved by the Human Science’s Research Council’s Research Ethics Committee and Human Subjects Review from the Centre for Disease Control and Prevention’s Global AIDS Programme. Dried blood spot (DBS) specimens were used for HIV antibody testing. An algorithm of three HIV enzyme immunoassays was used to test for HIV antibodies [6]. Full details of the survey methodology, including sample weighting, fieldwork procedures and quality control measures are described elsewhere [6], [8].

### Study Variables and Measures

Based on the multistage stratified sampling described above, this study draws on data collected from two groups of women aged 15–55: those who had been pregnant in the past two years and those interviewed as the parent or guardian of a child under 2 years. Data are drawn from four face-to-face questionnaires, specifically: a household-level questionnaire; a children below 2 years (reported by mother or guardian) questionnaire; a female youth aged 15–24 years questionnaire; and a women aged 25 to 55 years questionnaire.

Socio-economic quartiles (SEQ) were derived from measures of household-living standards, such as infrastructure and housing characteristics (source of drinking water, access to electricity, main source of energy for cooking, and type of toilet used) and household ownership of durable assets (presence of a working refrigerator, radio, television, cell phone and landline phone) captured in the household questionnaire. Quartiles were generated using multiple correspondence analysis [9], [10]. Socio-economic quartile groups were used instead of the more widely used quintiles because women overwhelmingly predominated in the poorer households, with few in the richer groups. For example, households in the 5th quintile contained only 61 (0.8%) of the total 8859 women aged 15 years and above, too low a frequency for meaningful analysis. Also, the socio-economic differentials between groups in rural communities are very narrow, given similar income-generation activities in these areas [11]. Hence, we deemed it most appropriate to use four socio-economic groups to differentiate households.

Study outcomes are drawn from two different study instruments: a health questionnaire completed by women aged 15–55 years who had been pregnant in the past two years (N = 1113), and women interviewed as the parent of a child born in the past two years (N = 1304). Only 632 women fell into both groups (only one respondent was selected for each questionnaire among all eligible household respondents). Women who had been pregnant in the past two years provided information on their general health status, whether their pregnancy had been planned, HIV testing in the past two years and their parity. Those who had a child under two gave data on their utilization of antenatal clinic services and delivery with a skilled birth attendant.

Survey instruments had not been specifically designed to measure maternal health status, thus available proxy indicators had to be used as measures of maternal health access and maternal health outcomes. Measures of access to health services were utilisation of antenatal clinics, HIV testing and having a skilled attendant at birth. In the absence of better indicators, having a doctor present at birth was included as a measure of health service access, even though interpretation of this indicator, like caesarean section rate, is not straightforward. The outcome HIV infection is included as a health status outcome, but we also examined whether there were systematic differences in access to services between those with and without HIV infection. Women responding with fair or poor to the question “In general, would you say that your health is excellent, good, fair or poor?”, were categorised as having a lower self-assessed health status and compared with those reporting good or excellent health. We included planned pregnancy and multiparity (five or more children) as measures of overall maternal health status, given their well-recognised links with health outcomes in pregnancy [12].

Distribution of access to services and of self-assessed health status was assessed across the following PROGRESS-Plus equity analysis groups: Place of Residence (province; locality as urban formal and informal, and rural formal and informal), Race, Occupation, Education, Socioeconomic Status (employment of the mother and being the household head), and age and HIV status representing the Plus category [13]. Maps were developed to show the distribution of antenatal and skilled birth coverage across districts of the country, using ArcGIS Desktop Version 10.0.

### Statistical Analysis

Data were analysed using Stata version 11.0 (College Station, Texas, United States), taking into account the complex multilevel sampling design and participant non-response. Weighting of the sample by age, race group and province was applied to ensure the study estimates are representative of the general population. Summary indices for descriptive analysis are weighted percentages, and unweighted counts are provided.

Clustering was not accounted for given that the large number of primary sampling units (1000) in the study is comparable to respondent number, diminishing such effects. Additionally, clustering at the household level was rare. Only 40 (3.6%) of the 1113 women, who had been pregnant in the past two years, were from the same household (one selected from women in the household 15–24 years and another from women 25–55 years). In univariable analysis, the distribution of maternal health outcomes across population groups were compared using the Rao-Scott F statistic to determine *P* values [14]. Multivariate logistic regression analysis, using backward fitting, was used to identify factors associated with utilisation of ANC before 20 weeks, SBA and having a doctor present at birth. These indicators of access to services were selected for further analysis as they have critical implications for outcomes of pregnancy and childbirth in this setting.

### Slope Index of Inequality, Relative Index of Inequality and Concentration Index

An absolute indicator of inequality (difference between QIV and QI) was calculated to measure inequalities in health access and status. Also, we used the slope index of inequality (SII) the relative index of inequality (RII) and the concentration index [15], [16]. These have the following desirable characteristics, they reflect: the socio-economic dimensions of health inequalities; the experience of the entire population rather than only that of Q1 and QIV; and changes in the distribution of the population across socio-economic groups [16]. SII is a measure of absolute effect, while the RII measures relative effects. Both measures are interpreted as the effect on health access or status of moving from the lowest to the highest socio-economic group (QI to QIV).

We followed standard methods for the calculation of equity indicators [15], [16]. Briefly, to calculate SII and RII, quartile groups were ordered from lowest to highest. The population of each quartile group is given a rank score based on the midpoint of its range in the cumulative distribution in the population. For example, biological mothers with four or more children in QI constituted 35.8% of the population, followed by 32.4% in the next highest quartile. QI was assigned a rank score of [0+ (0.358–0)/2] = 0.178, and next highest quartile a score of [0.358+ (0.680–0.358)/2) = 0.518 and so on. SII is then calculated as a weighted regression [16], of the health outcome and the rank of SEQ distribution, with weights as the number of individuals in the socio-economic quartile group. By weighting the quartile groups by their population share, the SII incorporates changes in the distribution of social groups’ that affect the population health burden of health disparities. The SII is the regression coefficient of the weighted regression model in Equation (1).
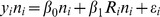
(1)Where 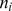 is the population size of QI, 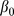 is the estimated health status of a hypothetical person at the bottom quartile and 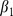, represents the SII, and is the absolute difference in health status between the bottom and top of the quartile, and 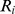 is the rank score. A unit change in relative rank is equivalent to moving from the bottom to the top of the quartile distribution.

RII is calculated using Equation (2), with 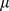 the population average of the specific health outcome.
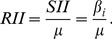
(2)


The concentration curve plots the cumulative proportion of health outcome against the cumulative proportion of the population, ranked by SEQ [17]. If health access is equally distributed across SEQ, concentration curves coincide with the diagonal line of equality. Concentration index- twice the area between the concentration curve and line of equality- ranges from –1 to 1. Zero represents perfect equality, while positive values indicate richer individuals have greater coverage (or worse health outcomes) than poorer individuals [17].

## Results

Of the 15,000 households sampled, only 13,440 were currently occupied; 80.8% of whom were interviewed (10,856/13,440). Non-response was largely due to refusal (9.3%, 1252/13,440) or no household member at home after four repeat visits (7.0%, 946/13,440). Of those interviewed 55.4% (9027/15,278) were women above 15 years, of whom 12.3% had been pregnant in the past two years (1113). Median time since giving birth was 11.0 months (inter-quartile range = 5.0–16.0 months).

### Characteristics of Women Pregnant in Last Two Years and their Socio-economic Status

Half the pregnant women were aged 20–29 years, another third were aged 30–39 years and only 0.5% were 50–55 years (Table 1). Overall 11.2% were under 20 years, though among the wealthiest quartile (QIV) only 4.5% were below 20. Scholars and students accounted for 7.6% of all pregnancies, with this proportion several fold higher among the bottom two than top two quartiles. Almost 90% of all pregnancies occurred in African women. Only 3.8% of women pregnant in the last two years were white, who made up 24.2% of QIV. Educational attainment was strongly linked with socio-economic status: as many as 59.9% of all women had not completed secondary school, but around half of QIV had tertiary education, 20-fold more than in QI. Most women reported being single (58.5%) and only 38.7% were married or cohabiting. In the lowest two quartiles, around 12% of pregnant women were also the household head, while only 7–9% were the household head within the higher two quartiles.

**Table 1:** Distribution of socio-economic status among population sub-groups of women pregnant in past two years: analysis of the 2008 national SABSSM survey.

		Socio-economic Quintile				
Variables/Categories		QI poorest (%)	QII (%)	QIII (%)	QIV wealthiest(%)	unweighted N OVERALL TOTAL %
**Age(years)**	**Categories**					N = 1103
	15–19	12	15.4	6	4.5	11.2
	20–29	51.7	47.9	54.4	47.9	50.8
	30–39	33.2	34.3	36.7	41.3	34.9
	40–54	3.2	2.4	3	6.3	3.1
**Place of residence**						N = 1107+
	Urban formal	8.9	44.4	85.2	97.3	43.3
	Urban informal	18.3	16.5	5.2	1.6	13.6
	Rural Informal	62.6	31.2	2.7	0.8	35.1
	Rural formal	10.3	7.9	6.9	0.3	8
**Province**						N = 1107+
	Western Cape	2.9	8.8	13.5	16.7	8.1
	Eastern Cape	18	7.3	11.3	14.6	12.9
	Northern Cape	1.2	2.4	2.3	0.9	1.8
	Free State	3.1	7.9	3.7	3.3	4.8
	KwaZulu-Natal	23.8	26.3	15.2	10.1	21.7
	North West	8.9	11.9	8.2	1.3	9.1
	Gauteng	9.1	18.2	40.9	42.8	21.4
	Mpumalanga	9.6	7.3	3.1	9	7.5
	Limpopo	23.4	9.9	1.8	1.2	12.8
**Race**						N = 1102+
	African	97.9	92.4	75.8	55.9	88.1
	White	0	0.1	8.6	24.2	3.8
	Mixed ancestry	2.1	6.8	14.4	13.5	7.1
	Indian	0	0.7	1.1	6.4	1
**Highest Education Level**						N = 1103+
	None or Grade 0–3	6.5	3.9	1.7	0	4.1
	Gr4-Gr7	17.2	6.5	6.9	2.9	10.5
	Gr8-Gr11	59	45.3	32.1	12.9	45.3
	Gr12	14.8	38.9	43.1	32.4	29.7
	Tertially	2.5	5.4	16.3	51.7	10.4
**Employment**						N = 1088+
	Housewife or Homemaker	25.7	17.8	15.2	11.2	19.8
	Unemployed, seeking work	41	36.4	33.4	29	37
	Unemployed, not seeking work	9.9	8.4	3	2.1	7.3
	Informal sector or self employed	5.1	4.8	5.7	15.9	6
	Student or Learner	7.9	11.4	3.5	2.5	7.6
	Formal sector part-time^∧^	5.4	4.3	5.1	2	4.7
	Formal sector full-time	3.5	14.9	34.1	37.2	16.3
	Other(pension,sick,disabled,other)	1.4	2.1	0	0.1	1.2
**Household Head**						N = 1082+
	Yes	12.4	12.6	7.8	9.2	11.3

Table shows column percentages. Only among women who had been pregnant in the past 2 years. *P* tests distribution of population group across quartiles;

+ *P*<0.05.

Only slightly more than a quarter of all women had any form of employment (27.0%). Even amongst the wealthiest quartile, 29.0% reported seeking work. Within the lowest quartile, only 14.0% were employed, with a mere 3.5% having full-time formal sector jobs. KwaZulu Natal accounted for 21.7% of all pregnant women, and also had the highest HIV prevalence, at 36.0% (Table S1). Pregnant women in the wealthiest quartile were heavily concentrated in the urban formal areas of Gauteng and Western Cape Province, as were the second wealthiest quartile. By contrast, almost two thirds of the poorest quartile lives in rural informal locations, the formal tribal areas of the country. This quartile lives mostly in the KwaZulu Natal (23.8%) and Limpopo (23.4%) provinces. The latter province has an especially high concentration of poor people, containing just a cumulative 4% of QIII and QIV.

### Inequities in Access to Maternal Health Services

**Figure 1:**
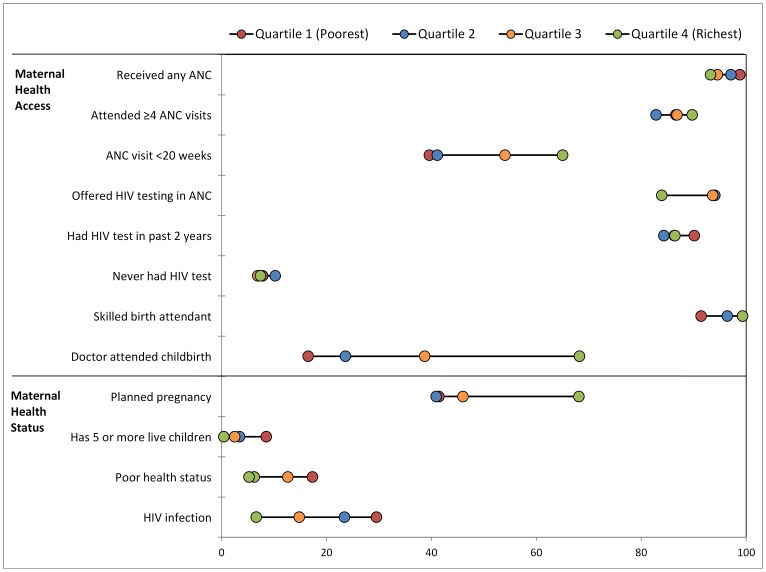
Differentials in coverage of maternal health services and in maternal health status in South Africa. Differentials in coverage of maternal health services and in maternal health status across socio- economic quartiles in South Africa.

**Figure 2:**
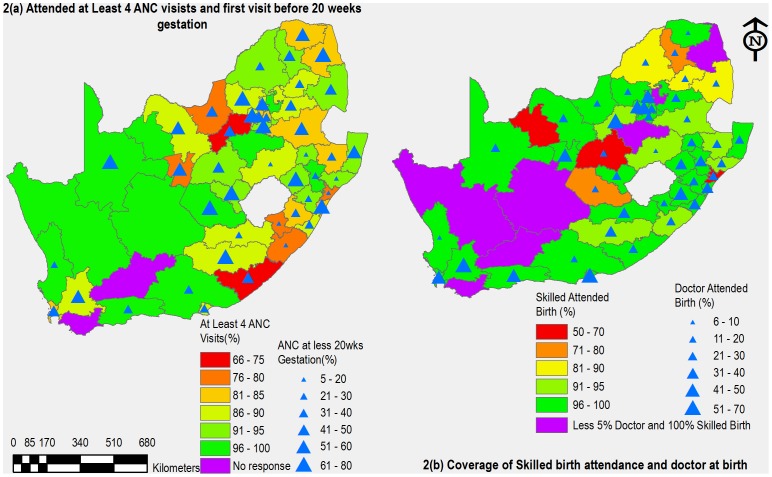
Utilzation of antenatal clinic services and skilled birth attendance in South Africa. Utilzation of antenatal clinic services and skilled birth attendance, by district in South Africa, findings of national survey.

Inequalities in access to key indicators are summarised in Table 2 and Figure 1 (further detail is provided in Table S1). Receipt of any ANC has the highest overall coverage and low levels of inequity, aside from notably lower levels in rural formal areas (89.7%; Table S1). A considerable portion of women, however, do not attend four antenatal visits, rural women in particular (Figure 2a). Moreover, fewer than half come before 20 weeks gestation, even lower among the African population, rural informal areas, those with minimal education and poorer women (C = 0.094). Poorest women had the highest ANC coverage (98.8%), but only 39.6% of them attended these services before 20 weeks gestation (Table 2). Early attendance was 2.7 fold higher in the wealthiest quartile (95%CI adjusted odds ratio [AOR] = 1.2–6.1; Table 3).

**Table 2:** Absolute and relative socio-economic inequalities in access to maternal health services and in maternal health status in South Africa: The 2008 national SABSSM survey.

		Socio-economic quartile (%)								
Variable category	Variable subcategory	QI poorest	QII	QIII	QIV wealthiest	OVERALL TOTAL% unweighted N	Difference (QIV-Q1) % points	Relative index of inequality (95%CI)	Slope index of inequality (95%CI)	ConcentrationIndex (C )
**Access to maternal health services**	**Received any ANC** ∧	98.8	97.1	94.5	93.2+	96.9 N = 1242	−5.6	−0.07 (−0.11 to −0.04)	−7.2 (−10.8 to −3.5)	−0.0115+
	**Attended ≥4 ANC visits** ∧	86.6	82.8	86.8	89.7	85.7 N = 1245	3.1	0.03 (−0.26 to 0.31)	2.2 (−21.9 to 26.3)	0.0039
	**ANC visit <20 weeks gestation** ∧	39.6	41.1	54.0	65.0+	45.2 N = 1244	25.4	0.63 (−0.35 to 1.61)	28.4 (−15.8 to 72.7)	0.0941
	**Offered HIV testing in ANC** ∧	93.8	94.0	93.6	83.9++	92.9 N = 1239	−9.9	−0.07 (−0.38 to 0.23)	−6.8 (−35.1 to 21.5)	−0.0112
	**Had HIV test in past 2 years** *	90.1	84.3	86.3	86.4	87.2 N = 1104	−3.7	−0.07 (−0.30 to 0.17)	−5.8 (−26.1 to 14.5)	−0.0101
	**Never had HIV test** *	7.8	10.2	6.9	7.4	8.3 N = 1104	−0.4	−0.10 (−1.85 to 1.65)	−0.8 (**−**15.4 to 13.7)	**−**0.0141
	**Skilled birth attendant** ∧	91.4	96.4	99.3	99.3+	95.3 N = 1257	7.9	0.12 (0.04 to 0.20)	11.6 (3.8 to 19.5)	0.0122
	**Doctor attended childbirth** ∧	16.5	23.6	38.7	68.2+	27.7 N = 1257	51.7	1.94 (**−**1.03 to 4.90)	52.3 (**−**27.7 to 132.2)	0.2694+
**Maternal health status**	**Planned pregnancy** *	41.4	40.9	46.0	68.1+	44.4 N = 1097	26.7	0.44 (**−**1.01 to 1.90)	19.6 (**−**45.1 to 84.3)	0.0665
	**Has 5 or more live children** *	8.5	3.4	2.5	0.4+	5.0 N = 1005	**−**8.1	**−**2.09 (**−**3.89 to **−**0.29)	**−**10.2 (**−**1.4 to 19.0)	**−**0.3319+
	**Poor-fair health status** *	17.3	12.6	6.2	5.2+	12.5 N = 1105	**−**12.1	**−**1.35 (**−**1.97 to **−**0.73)	**−**16.8 (**−**24.6 to **−**9.1)	**−**0.2106
	**HIV infection** *	29.5	23.4	14.8	6.6+	23.0 N = 890	**−**22.9	**−**1.13 (**−**1.98 to **−**0.27)	**−**25.9 (**−**45.6 to **−**6.2)	**−**0.1755

* In women who had been pregnant in the past 2 years.

∧ In women who had a child in past two years. *P* tests distribution of outcome across quartiles;

+ *P*<0.05;

++ *P≥*0.05 & *P*<0.1. Poor-fair health status is self-assessed.

**Table 3:** Multivariate logistic regression analysis of factors associated with access to maternal health services in South Africa: Early antenatal attendance, skilled birth attendance and having a doctor present at childbirth.

	ANC visit <20 weeks		Skilled birth attendant		Doctor at childbirth	
Population group	Unadjusted OR (95%CI)	Adjusted OR (95%CI)	Unadjusted OR (95%CI)	Adjusted OR (95%CI)	Unadjusted OR (95%CI)	Adjusted OR (95%CI)
**Age (years)**						
15–19	1.0		1.0		1.0	1.0
20–29	0.88 (0.58–1.35)		0.93 (0.35–2.47)		1.14 (0.68–1.92)	1.26 (0.52–3.05)
30–39	1.03 (0.62–1.71)		1.87 (0.53–6.63)		1.60 (0.93–2.78)	1.42 (0.60–3.36)
40–54	1.81 (0.79–4.14)		0.71 (0.13–3.71)		1.31 (0.50–3.43)	0.79 (0.15–4.16)
**Place of residence**						
Rural informal	0.61 (0.40–0.91)	0.86 (0.55–1.34)	0.11 (0.03–0.37)	0.18 (0.04–0.93)	0.35 (0.23–0.55)	0.93 (0.48–1.80)
Rural formal	0.92 (0.52–1.64)	1.21 (0.62–2.34)	0.05 (0.01–0.17)	0.07 (0.01–0.33)	0.55 (0.28–1.08)	0.64 (0.28–1.42)
Urban informal	0.91 (0.57–1.44)	1.18 (0.73–1.92)	0.18 (0.04–0.74)	0.12 (0.02–0.66)	0.77 (0.47–1.25)	1.35 (0.69–2.62)
Urban formal	1.0	1.0	1.0	1.0	1.0	1.0
**Socio-economic quartile**						
I	1.0	1.0	1.0	1.0	1.0	1.0
II	1.07 (0.73–1.56)	1.03 (0.69–1.52)	2.51 (1.06–5.95)	1.55 (0.56–4.30)	1.57 (1.04–2.35)	1.13 (0.66–1.91)
III	1.79 (1.12–2.87)	1.66 (0.99–2.79)	13.24 (2.13–82.09)	15.29 (1.81–129.17)	3.20 (1.96–5.22)	1.51 (0.69–3.28)
IV	2.84 (1.32–6.14)	2.68 (1.17–6.13)	14.25 (1.84–110.36)	0.15 (0.01–2.58)	10.85 (4.04–29.12)	4.84 (1.60–14.67)
**Race**						
African	1.0		1.0		1.0	1.0
Mixed ancestry	1.53 (0.97–2.40)		1.57 (0.35–7.04)		1.93 (1.20–3.09)	1.13 (0.54–2.37)
Indian	2.46 (1.17–5.18)		#		10.03 (4.81–20.90)	12.06 (3.74–38.84)
White	2.10 (0.72–6.14)		#		6.66 (1.33–33.3)	24.59 (4.77–126.65)
**Highest maternal education**						
None or Grade 0–3	1.0		1.0	1.0	1.0	1.0
Grade 4–7	1.31 (0.50–3.48)		1.86 (0.45–7.74)	3.28 (0.79–13.83)	0.21 (0.56–0.79)	0.22 (0.05–0.96)
Grade 8–11	1.21 (0.53–2.75)		2.13 (0.63–7.12)	2.55 (0.77–8.45)	0.42 (0.167–1.05)	0.44 (0.15–1.26)
Grade 12	1.95 (0.82–4.61)		97.53 (10.52–904.10)	95.51 (10.41–875.88)	1.70 (0.68–4.25)	1.32 (0.47–3.70)
Tertiary	0.98 (0.30–3.17)		37.88 (3.89–369.35)	45.55 (2.28–909.53)	5.05 (1.67–15.27)	2.18 (0.67–7.12)
**Employment**						
Unemployed, seeking work	0.75 (0.46–1.23)		2.10 (0.78–5.66)		1.29 (0.69–2.40)	
Unemployed, not seeking work	0.49 (0.22–1.09)		6.20 (0.76–50.64)		0.65 (0.26–1.61)	
Housewife or homemaker	1.0		1.0		1.0	
Student or learner	0.69 (0.32–1.50)		4.64 (0.56–38.61)		0.84 (0.30–2.36)	
Informal sector, self employed	2.69 (1.10–6.59)		1.63 (0.30–8.75)		2.15 (0.82–5.66)	
Formal sector part–time	0.57 (0.22–1.43)		12.63 (1.51–105.50)		6.66 (3.23–13.73)	
Formal sector full–time	1.10 (0.53–2.28)		8.39 (1.02–69.03)		0.72 (0.24–2.18)	
Other (disabled, sick, other)	#		#		1.61 (0.18–14.77)	
**Marital status**						
Single	1.0		1.0		1.0	
Married/cohabiting	1.31 (0.86–1.98)		1.10 (0.46–2.64)		1.54 (0.95–2.50)	
Widowed or divorced	2.20 (0.67–7.27)		0.44 (0.05–3.69)		1.58 (0.55–4.57)	

In women who had a child in past 2 years. OR odds ratio; CI confidence interval.

# Category omitted as no failures occurred in group.

Minimal SES inequalities were noted in all three measures of HIV testing; with testing levels slightly higher among the poorest (C for all three measures = –0.01; Tables 2, Figure 1). Being offered HIV testing was highest among the poorest quartile (93.8%), compared to only 83.9% of the wealthiest. Testing in the past two years differed by 5.8 percentage points (SII = –5.8), with a relatively higher decrease (7%, RII = –0.07) among those in the top quartile (Table 2). A step-wise reduction in likelihood of being offered HIV testing was noted with each increase in age group. Fully 17.6% of women 40–54 years had never tested. Discordance between levels of HIV testing being offered and uptake were seen in women under 20∶96.1% were offered HIV testing, but 12.2% had never tested. Markedly fewer women in rural formal areas had been offered HIV testing, a population group with under-servicing detected on several measures. Almost 95% of all African and Mixed Ancestry women were offered HIV testing in pregnancy, though test uptake appears higher in Mixed Ancestry women. In this group, 3.2% had never had an HIV test, compared to 8.9% of African women. Nearly 10% of women who were HIV infected reported not having been offered HIV testing during pregnancy, and 6.7% said they had never tested. Overall testing coverage was lowest in Mpumalanga, Limpopo and Eastern Cape provinces (15.7%, 10.4 and 9.1% correspondingly had never had a test) and in those with low levels of education (Table S1).

Inequalities were noted in SBA, with several population groups well below the population average of 95.3%. Only 91.4% of the poorest quartile had an SBA, while coverage was near universal among the wealthiest two quartiles. There was a considerable percentage difference of 11.5 (SII = 11.6) in SBA from QI to IV (Table 2). In multivariate analysis, controlling for socio-economic status and locality, very sizable associations were detected between level of education and SBA coverage, especially among those who had completed secondary or tertiary education (Table 3). In the same multivariate model, compared with urban formal areas, SBA coverage was 82–93% less likely in the rural informal, urban informal or rural formal areas. Specifically, women in rural formal areas were 93% less likely to have a SBA than urban formal women (95%CI = 67%–99%). Within 7 of the 52 districts in South Africa, SBA coverage was below 86% (Figure 2b). Conversely, in 30 other districts, coverage was above 98%. Coverage was 100% in all districts of the Western Cape Province, while poorly-performing districts were mostly located within the provinces of Limpopo, Mpumalanga, Free State and KwaZulu Natal (Figure 2b). Though high SBA coverage and doctors present at birth were correlated, it is important to note that in seven districts (Namakwa, Overberg and Central Karoo in Western Cape; Pixley ka Seme in Northern Cape; Northern Free State in Free state, Metsweding in Gauteng and Mopani in Limpopo) fewer than 5% had a doctor at birth and yet still SBA levels were 100% (Figure 2b). Of concern, ILembe District Municipality in Kwazulu Natal had the lowest skilled birth attendance (50.2%), and notably it is 92.8% rural informal, 77.7% of mothers are household heads and 100% had not completed secondary school.

Based on all measures in Table 2, doctor attending childbirth was the most unequal of all study outcomes (concentration index = 0.269), with an increase of 194% among women in the top quartile (RII = 1.94). Associations were detected between most PROGRESS-Plus groups and this outcome (Table S1). Around two thirds of women in the highest quartile had a doctor present at childbirth, 10.9-fold higher than the poorest quartile. In multivariate analysis, adjusting for age, place of residence, socio-economic status and education, White and Indian women were 10–20 fold more likely to have a doctor present at childbirth than African women.

### Distribution of Planned Pregnancy and Multiparity

Overall, only 44.4% of pregnancies were planned, lowest in KwaZulu Natal (25.5%) and Eastern Cape provinces (38.1%). Levels were clustered around a similar range for the lower three quartiles, but 68.1% of the wealthiest quartile had a planned pregnancy (Table 2). Almost 90% of pregnancies in those aged under 20 were unplanned, while 57.3% were planned among women 30–39 years. Among women with HIV, only 31.7% of pregnancies were planned, compared with 42.1% of those non-infected (*P* = 0.07). Planned pregnancy was also less common among the 11.7% of women who already had more than four children (32.7%), compared to 45.5% of other women (*P* = 0.04). There was a step-wise marked decrease in the proportion that had more than four children with each increase in education level or wealth quartile (Table S1). Only 8.3% of women in formal urban areas had four or more children, compared with 19.0% of women in rural formal areas.

### Inequities in Maternal Health Status, and Associations between HIV and Study Outcomes

Large differentials were noted in self-assessed health status across essentially all PROGRESS-Plus groups (Table 4). Poor-fair health status was concentrated among the poorest (C = –0.21.6; Table 2). Self-assessed health status declined considerably with each drop in socio-economic quartile, education level or age group. A fifth to a sixth of women who were household heads or lived in rural areas assessed their health as poor-fair. By contrast, for nearly fifteen other population groups, fewer than 10% described their health status as only fair or poor. A quarter of HIV-infected women described themselves as having poor or only fair health. More than a third of women in KwaZulu Natal and Mpumalanga Provinces were HIV infected, and in these provinces nearly 20% of women reported having poor-fair health.

**Table 4:** Absolute percentage differences in maternal health access and health status across different populations groups in South Africa: The 2008 national SABSSM survey.

	Percent difference between highest and lowest groups in access to maternal health services								Percent difference between highest and lowest groups in maternal health status			
Population group	Receivedany ANC∧	Attended ≥4 ANC visits∧	ANC visit <20 weeks∧	Offered HIV test in ANC∧	HIV test in past 2 years*	Never had HIV test*	Skilled birth attendant∧	Doctor at childbirth∧	Planned pregnancy*	Has 5 or more live children*	Poor-fair health status*	HIVinfection
**Age group**	4.8	4.2	17.8	13.9+	15.7+	11.4	4.0	9.4	36.4+	28.8+	12.7+	16.8+
**Place of residence**	9.8+	9.5	12.3+	10.8++	4.3	3.8	13.6+	19.9+	19.0+	4.5+	8.0+	20.1+
**Province**	5.5	14.0	37.4++	9.7	12.5	12.1	13.8+	31.4+	30.9+	8.2	12.4++	25.6+
**Race**	24.0+	20.4++	22.0++	33.1+	15.6	6.8+	3.4	52.0+	4.0+	5.5	12.0	25.9+
**Maternal education level**	11.8++	1.8	16.7	8.9	11.5	13.0+	10.8+	61.5+	34.0+	28.8+	37.5+	29.4+
**Employment status**	13.0+	33.5+	71.5+	13.6	13.2	8.9	8.2	49.6+	48.5+	10.0+	64.6+	30.7+
**Household head**	3.2	4.7	2.2	1.1	1.2	1.9	1.5	8.5	8.7	5.1+	9.8+	4.5
**HIV infection**	2.0+	5.9	0.7	2.2	3.2	1.9	8.3+	9.8++	10.4++	1.0	16.4+	–

* Among women who had been pregnant in past 2 years.

∧ In women who had a child in past 2 years.

+ *P*<0.05;

++ *P*≥0.05 & *P*<0.1. Poor-fair health status is self-assessed.

Distribution of HIV infection among population groups is markedly unequal, with double digit absolute percentage differentials between all PROGRESS-Plus groups (range = 16.8–30.7% (Table S1), apart from household head. African women had a 25.9% HIV prevalence, compared to 3.5% of Mixed Ancestry women and no infections in the other groups. Socio-economic differentials in levels of HIV infection were marked: C = –0.1755 and SII = –25.9.

Among HIV-infected women, attendance at antenatal clinic was near universal and 90.6% had at least four ANC visits. However, only 46.2% had their first visit before 20 weeks of pregnancy. Of women with HIV infection, only 17.0% had a doctor present at childbirth, compared to 26.8% of other women (*P* = 0.06). Also, concerningly, fewer HIV-infected women had an SBA than those non-infected (88.0% versus 96.3%; *P* = 0.03; Table S1).

## Discussion

Using data available from a household survey designed primarily for other purposes, we have been able to examine inequalities in maternal health care service access and outcomes. Aside from early attendance at antenatal clinic and deficiencies in overall access in some provinces and rural formal areas, inequalities in utilisation of maternal health services were mostly small. Several measures were even higher amongst the poorest quartile. Considerably larger differences, however, were noted between measures of maternal health status across population groups. This may reflect variations between the PROGRESS-Plus groups in the quality of care received, burden of HIV infection and in social determinants of health. In particular, disparities in education level and employment, for example, were marked between wealth quartiles and provinces of the country.

Differentials in coverage of services must also be interpreted in light of markedly asymmetric needs between population groups, as measured by HIV status, for example. HIV infection is the preeminent risk factor for maternal morbidity and mortality in the country [3], and these women require more, not equal, levels of services than others. Therefore, in particular, poorly-educated women, or those in rural informal or urban informal areas, have very high levels of need, as measured by HIV status. Notably, women in rural formal areas had the lowest levels of many indicators, specifically: ANC coverage, completion of four ANC visits, offered HIV testing and skilled birth attendant present at childbirth. Future research should examine these geographical inequities in more detail, and government should focus their efforts on improving access to high-quality services in these areas. This would involve identifying districts and communities where access is a problem, and disaggregating the relative contribution of factors accounting for this, such as lack of service availability or quality, poor patient experiences and perceived poor quality of services. Finally, women living within female-headed households, which have about half the annual income of male-headed households [18], are twice as likely to have five or more children or to report poor-fair health status as women within male-headed households.

Enhanced family planning services, with a reduction in unplanned pregnancies, would lower maternal mortality, and is also one of four strategies promulgated globally to reduce mother-to-child transmission (MTCT) of HIV [18]. Though there is a recent global upsurge in attention paid to family planning [19], these health service are long neglected, and appear largely ineffective in South Africa, with nearly 70% of pregnancies among HIV-infected women being unplanned. Growing resources for family planning present a critical opportunity for reaching the planned MTCT elimination targets. Also, the substantial variation in planned pregnancy and multiparity across population groups demonstrates a need to focus on reaching vulnerable groups in addition to those with HIV. Unmet need for family planning services appears most acute among women who are poor or did not complete primary school, and those in rural informal areas, especially of KwaZulu Natal and Eastern Cape. Encouragingly, in South Africa, teenage pregnancy rates have declined for some years [20], consistent with relatively low rates noted in this study.

Seemingly, there are several demand-side barriers to early attendance at ANC, with overall levels low, but especially marked among women who are poor, black or live in rural informal areas. Presently, women have little incentive to attend ANC early in pregnancy. Late and incomplete attendance have major health consequences, especially for women infected with HIV who often require several visits during pregnancy to initiate the necessary drugs to secure their health and to ensure their child is not infected with HIV. Moreover, as antiretroviral drugs are now widely available [21], the most critical factor which determines whether a woman transmits HIV to her infant is the duration of antiretroviral therapy during pregnancy [22], [23]. Each additional week during pregnancy that a woman takes these drugs is vital. Equally, with foetal alcohol spectrum disorders, there are tremendous benefits to early antenatal attendance with alcohol screening and interventions, as well as for repeat visits with reinforcement of the need for alcohol abstinence. Levels of this condition are extremely high in Western and Northern Cape, and more generally in rural areas and those of mixed ancestry [24], [25]. Targeted efforts to optimise early ANC utilisation are needed in areas heavily-affected by this condition.

Access to HIV testing is high, especially among the poorest, though coverage is suboptimal in Limpopo and Mpumalanga provinces, and in those with very limited education. Importantly, about 15% of HIV-infected women who had been pregnant in the past two years had not had an HIV test and nearly 10% were not offered testing during pregnancy. Differentials in being offered HIV testing in ANC, which should be universally provided in South Africa [26], warrant further investigation. This intervention has low coverage among older women (almost a fifth had never tested) and some race groups. It is possible that low HIV testing rates among older women reflects difficulties of an often considerably younger peer counsellor in discussing HIV testing and sexual matters with older women. Low testing rates in some races might also reflect difficulties in some races counselling another race. The PMTCT programme in the country could consider of policy of recruiting HIV counsellors with a mix of ages and race.

Similar to our finding of strikingly low SBA coverage in some districts of the country, analyses from demographic surveillance sites in rural areas calculated the proportion of home births as 64% between 2000 and 2007 for the Hlabisa area, and 23% between 2000 and 2005 for Agincourt, while this was an estimated 50% for a hospital in the Eastern Cape in 2005 [4], [27]. The Demographic and Health Survey in 2003 also found 27% of women delivered with a doctor [2], the same as in this study.

There are significant differentials in access to a doctor performing the delivery though this indicator is difficult to interpret. National guidelines recommend low-risk births be managed by midwives and that only about 10% of women require the services of a specialist obstetrician at childbirth [28]. High proportions in wealthy women with health insurance may indicate over-servicing and health system inefficiencies [28], [29]. However, in some provinces of the country and among women in the lowest socio-economic quartile, only about 15% had a doctor present at childbirth. This might reflect a lack of access to secondary or tertiary levels of care for women in these groups who require such services during childbirth. Interestingly, Limpopo and Mpumalanga provinces, with the lowest levels of doctor at birth, had much higher levels of maternal deaths due to anaesthetic complications than other provinces in a national maternal death review [3]. In that review, the Free State province had the highest institutional maternal mortality ratio (MMR) of all provinces, while in this study it has among the lowest SBA and doctor coverage at birth. Conversely, high doctor and SBA coverage was detected in the largely urban and wealthiest provinces of the Western Cape and Gauteng, which have the lowest institutional MMR and deaths due to anaesthetic complications. Also, an association was detected between the proportion of women at district level who attended ANC before 20 weeks, had a skilled birth attendant or had a doctor present at childbirth and the district-level institutional MMR in the national report.

## Limitations

This study reflects the benefits, and the limitations, of secondary data analyses. Some of the measures of maternal health status used in this secondary analysis have limited construct and content validity, especially planned pregnancy and multiparity, which are only indirect indicators of maternal health. Aside from biological measures of HIV status, the study outcomes were self-reported, and may be subject to social desirability and recall biases. Misclassification biases may be especially marked with the variable self-assessed health status. Though previous studies have shown this measure is associated with morbidity and predicts mortality, there may be some variation in the validity of this indicator across population groups [30], [31], [32]. Studies on maternal health inequity in this country require better indicators of health status such haemoglobin levels, as well as indicators of the quality of maternal care received, not only utilisation. The demographic and health survey (DHS) questionnaire addresses some of these limitations, but there has been no DHS survey in South Africa since 2003. In the meantime, additions could be made to the tool used in the survey reported here in order to evaluate this wider range of objectives.

The particular socio-economic profile of the country, with women-headed households and large proportions of the population engaged in similar economic activities, means that quartiles were preferred, rather than quintiles as in most equity studies. This limits comparability between studies.

As with all cross-sectional data, we are unable to infer causality between, for example, access to services and health outcomes. Similarly, though self-assessed health status within two years of childbirth is arguably a valid proxy of maternal health, clearly this status may be influenced by factors other than health during pregnancy, childbirth and the postpartum (though women were only a median 11 months postpartum).

The proportion declining participation in the main survey is substantial. Of particular concern, it is plausible that the decision to decline enrolment may be associated with key study outcomes. For example; those who decline participation or HIV testing may have different health-seeking behaviours than those who participate. Moreover, wealthier and white race groups were more likely to decline survey participation, potentially introducing bias as these groups have a lower HIV prevalence than included groups [6]. These groups may also incur specific forms of measurement bias. For example, wealthy women who visited a private-sector obstetrician during pregnancy may have reported not having attended an antenatal clinic during pregnancy.

Though place of residence provides a measure of geographical access, the study was unable to adequately control for possible effects of the distance between a household and health facility on service use. Finally, data were not available in the survey for all PROGRESS-Plus groups, notably absent for religion, social capital and for health insurance status.

## Conclusions

Despite the limitation of using data from a household survey primarily designed for other purposes, this analysis has provided useful information on inequalities in maternal health care service access and outcomes. Most striking is the intensely skewed distribution of the burden of HIV infection and assessment of poor-fair health status across population groups. These findings are likely accounted for by differentials in social determinants of health and in the quality of care received, given that the levels of access to services were broadly similar across groups. Though the health system has a critical role to play in narrowing some gaps in access noted and in improving quality of care, factors outside the health sector are also key to determining health status of the population and the marked differentials noted here.

Gaps in the health outcomes measured were considerable, with large step-wise reductions in health status across socio-economic groups, for example. Though differentials in access to some maternal health services were detected, for many indicators such differences were relatively small, even benefitting the poorest women in a few instances. For the poorest quartile, attendance at ANC is high, but they attend later in pregnancy than wealthier women, and have much lower skilled birth attendance levels.

In terms of service access gaps to further reduce MTCT of HIV, we recommend the government prioritise reductions in unplanned pregnancies among HIV-infected women; promotion of visits to ANC early in pregnancy especially in some key population groups; and the targeting of areas that still have low HIV testing coverage. Moreover, for reducing overall inequities in maternal health care service access, concerted efforts are required in some districts of the country to raise access to key maternal health interventions, particularly early ANC attendance, having four ANC visits and access to a skilled birth attendant at delivery.

Lastly, improvements in the national health information system are required to routinely monitor the level and equity of access to quality maternal health services and outcomes. A number of these indicators can only be measured in regular household health surveys specifically designed to capture maternal health status.

## Supporting Information

Table S1 Inequities in maternal health across different populations groups in South Africa: analysis of the 2008 national SABSSM survey.
(DOCX)
