# Supplementary material for: Equity in Maternal Health in South Africa: Analysis of Health Service Access and Health Status in a National Household Survey
Source: PLoS One. 2013 Sep 6;8(9):e73864. doi: 10.1371/journal.pone.0073864 (PMC3765324; doi:10.1371/journal.pone.0073864)
Supplement: Table S1 — Inequities in maternal health across different populations groups in South Africa: analysis of the 2008 national SABSSM survey. (DOCX) [file pone.0073864.s001.docx]

**Table S1. Inequities in maternal health across different populations groups in South Africa: analysis of the 2008 national SABSSM survey**

|  | **Level of access to maternal and reproductive health services (%)** | | | | | | | | | **Maternal health status (%)** | | |
| --- | --- | --- | --- | --- | --- | --- | --- | --- | --- | --- | --- | --- |
| **Population group** | **Planned pregnancy*** | **Received any ANC^** | **Attended ≥4 ANC visits^** | **ANC visit <20 weeks^** | **Offered HIV test in ANC^** | **HIV test in past 2 years*** | **Never had HIV test*** | **Skilled birth attendant^** | **Doctor at childbirth^** | **Has 5 or more live children*** | **Poor health status*** | **HIV infection** |
| **Age (years)** |  |  |  |  |  |  |  |  |  |  |  |  |
| 15-19 | 12.2^+^ | 100 | 82.9 | 45.9 | 96.1^+^ | 87.5^+^ | 12.2 | 95.1 | 23.2 | 0.0^+^ | 7.9^+^ | 11.6^+^ |
| 20-29 | 42.1 | 96.7 | 87.1 | 42.8 | 94.7 | 90.7 | 6.2 | 94.8 | 25.6 | 1.4 | 9.1 | 22.6 |
| 30-39 | 57.3 | 95.2 | 84.5 | 46.7 | 89.5 | 83.5 | 9 | 97.3 | 32.6 | 8.7 | 18.1 | 28.4 |
| 40-54 | 48.6 | 100 | 87 | 60.6 | 82.2 | 75 | 17.6 | 93.3 | 28.4 | 28.8 | 20.6 | 16.1 |
| **Place of residence** |  |  |  |  |  |  |  |  |  |  |  |  |
| Rural informal | 56.2^+^ | 99.5^+^ | 85.2 | 38.2^+^ | 94.8\| | 88.6 | 9.4 | 92.9^+^ | 17.0^+^ | 6.7^+^ | 16.6^+^ | 25.2^+^ |
| Rural formal | 37.2 | 89.7 | 79.7 | 48.4 | 84 | 84.3 | 5.6 | 85.6 | 24.3 | 5.7 | 15.3 | 16.9 |
| Urban informal | 41.2 | 97 | 89.2 | 48 | 94 | 88.3 | 5.7 | 95.4 | 31 | 7.1 | 12.9 | 37 |
| Urban formal | 48.5 | 95.9 | 86.5 | 50.5 | 92.3 | 86.4 | 8.6 | 99.2 | 36.9 | 2.6 | 8.6 | 17.5 |
| **Province** |  |  |  |  |  |  |  |  |  |  |  |  |
| Western Cape | 43.2^+^ | 94.7 | 84.7 | 47.3\| | 92.1 | 88.3 | 3.6 | 100^+^ | 42.4^+^ | 0.0 | 10.9\| | 10.4^+^ |
| Eastern Cape | 38.1 | 98.3 | 79.5 | 30.8 | 96.9 | 88.3 | 9.1 | 96.3 | 28.4 | 4.3 | 13.2 | 18.6 |
| Northern Cape | 49 | 99.4 | 93.5 | 68.2 | 98.4 | 94.4 | 4.1 | 99.4 | 21.7 | 8.2 | 12.3 | 11.7 |
| Free State | 51 | 98.4 | 92.9 | 49.6 | 93.8 | 89 | 4.8 | 86.2 | 19.5 | 1.9 | 7.8 | 10.4 |
| KwaZulu-Natal | 25.5 | 97.1 | 88.7 | 42.6 | 94.7 | 88.7 | 7.9 | 94.9 | 29.5 | 6.0 | 19.3 | 36 |
| North West | 51.8 | 95.3 | 84 | 52.8 | 90.2 | 88.2 | 5.8 | 96 | 25.5 | 1.5 | 10.8 | 18.3 |
| Gauteng | 56.4 | 94.5 | 83.4 | 44.2 | 91.1 | 85.2 | 8.2 | 98.6 | 36.4 | 5.5 | 9.1 | 23.1 |
| Mpumalanga | 46.1 | 98.2 | 89.1 | 48.5 | 91.9 | 81.9 | 15.7 | 93.9 | 18 | 7.5 | 19.3 | 34.5 |
| Limpopo | 52 | 100 | 87.3 | 55.1 | 88.7 | 86.8 | 10.4 | 90.8 | 11 | 6.9 | 6.9 | 18.5 |
| **Race** |  |  |  |  |  |  |  |  |  |  |  |  |
| African | 42.8^+^ | 98.3^+^ | 85.9\| | 43.1\| | 94.8^+^ | 87.7 | 8.9^+^ | 94.8 | 23.5^+^ | 5.5 | 13 | 25.9^+^ |
| Coloured | 39 | 97.4 | 92.5 | 53.6 | 94.9 | 90.3 | 3.2 | 96.6 | 37.3 | 1.9 | 7.9 | 3.5 |
| Indian | 57.9 | 89.8 | 82 | 65.1 | 70.8 | 83.1 | 8.7 | 100 | 75.5 | 0.0 | 1 | 0 |
| White | 80 | 74.3 | 72.1 | 61.4 | 61.8 | 74.7 | 2.1 | 100 | 67.2 | 0.0 | 7.8 | 0 |
| **Highest maternal education** |  | 0.07 |  |  |  |  |  |  |  |  |  |  |
| None or Grade 0-3 | 27.7^+^ | 92.1 | 85.8 | 35.7 | 90.5 | 81.6 | 14.5^+^ | 89.1^+^ | 32.2^+^ | 28.8^+^ | 40.2^+^ | 42.7^+^ |
| Grade 4-7 | 43.1 | 97.6 | 84.5 | 42.2 | 96.5 | 93.1 | 4 | 93.8 | 9.1 | 14.2 | 17.2 | 20.2 |
| Grade 8-11 | 38.3 | 98.6 | 84.2 | 40.3 | 94.6 | 84.9 | 10.5 | 94.5 | 16.6 | 4.6 | 12.3 | 29 |
| Grade 12 | 49.2 | 96.6 | 86 | 52 | 93.4 | 88.3 | 8 | 99.9 | 44.7 | 0.3 | 11.1 | 14.1 |
| Tertiary | 61.7 | 86.8 | 85.6 | 35.3 | 87.6 | 90.7 | 1.5 | 99.7 | 70.6 | 0.0 | 2.7 | 13.3 |
| **Employment** |  |  |  |  |  |  |  |  |  |  |  |  |
| Unemployed, seeking work | 33.5^+^ | 98.7^+^ | 90.8^+^ | 41.2^+^ | 96.4 | 87.1 | 8.3 | 95.9 | 26.4^+^ | 6.3^+^ | 12.1^+^ | 30.8^+^ |
| Unemployed, not seeking work | 31.8 | 99.2 | 75 | 31.2 | 96.3 | 91.9 | 8.1 | 98.6 | 15.4 | 1.5 | 8.6 | 16.5 |
| Housewife or homemaker | 62 | 96.5 | 87.3 | 48.3 | 91.7 | 86.9 | 8.3 | 91.8 | 21.8 | 10.0 | 12.2 | 24.6 |
| Student or learner | 13.9 | 100 | 66.5 | 39.3 | 94.7 | 88.9 | 8.8 | 98.1 | 18.9 | 0.0 | 6.1 | 11 |
| Informal sector, self employed | 58.8 | 99.1 | 91.7 | 71.5 | 95.6 | 87.2 | 2.6 | 94.8 | 37.5 | 5.1 | 15.5 | 31.3 |
| Formal sector part-time | 50.4 | 99.5 | 93.8 | 34.6 | 95.3 | 90.2 | 7.2 | 99.3 | 16.7 | 1.0 | 19.2 | 9.8 |
| Formal sector full-time | 62.9 | 87 | 75 | 50.7 | 86.4 | 85 | 9.8 | 98.9 | 65 | 0.3 | 10 | 10.3 |
| Other (disabled, sick, other) | 14.4 | 100 | 100 | 0 | 100 | 78.7 | 11.5 | 100 | 31 | 6.4 | 70.7 | 40.5 |
| **Household head** |  |  |  |  |  |  |  |  |  |  |  |  |
| Yes | 52.1 | 99.8 | 89.3 | 41.9 | 94.9 | 88.1 | 6.7 | 94.5 | 21.2 | 9.3^+^ | 21.4^+^ | 27.4 |
| No | 43.4 | 96.6 | 84.6 | 44.1 | 93.8 | 86.9 | 8.6 | 96 | 29.7 | 4.2 | 11.6 | 22.9 |
| **HIV** | 0.07 |  |  |  |  |  |  |  | 0.06 |  |  |  |
| Infected | 31.7 | 99.1^+^ | 90.6 | 46.2 | 91.3 | 85.1 | 6.7 | 88.0^+^ | 17 | 5.4 | 25.1^+^ | - |
| Non-infected | 42.1 | 97.1 | 84.7 | 45.5 | 93.5 | 88.3 | 8.6 | 96.3 | 26.8 | 4.1 | 8.7 |  |

*Only among women who had been pregnant in past 2 years. ^In women who had a child in past 2 years. ^+^*P*<0.05 | *P≥*0.05 & *P*<0.1
